# A rare case of right heart failure with the necessity for veno‐arterial extracorporeal membrane oxygenation following pulmonary vein stenosis after radiofrequency ablation for atrial fibrillation

**DOI:** 10.1002/pul2.12189

**Published:** 2023-02-21

**Authors:** Sabrina Kopp, Marie‐Kristin Tilch, Ingo Sagoschen, Joachim Kaes, Malte Kuniss, Thomas Neumann, Yang Yang, Katharina Schnitzler, Kai‐Helge Schmidt, Thomas Rostock, Thomas Münzel, Stavros Konstantinides, Johannes Wild, Lukas Hobohm

**Affiliations:** ^1^ Department of Cardiology, Cardiology I University Medical Center Mainz Mainz Germany; ^2^ Department of Hematology, Oncology and Pneumology & University Cancer Center University Medical Center Mainz Mainz Germany; ^3^ Department of Cardiology Kerckhoff Heart Center Bad Nauheim Germany; ^4^ Department of Radiology University Medical Center Mainz Mainz Germany; ^5^ Center for Thrombosis and Hemostasis (CTH) University Medical Center Mainz Mainz Germany

**Keywords:** catheter ablation, extracorporeal membrane oxygenation, pulmonary hypertension, pulmonary vein stenosis, right heart failure

## Abstract

Pulmonary vein stenosis (PVS) after radiofrequency energy‐mediated percutaneous pulmonary vein isolation as a treatment option for atrial fibrillation is a serious complication and the prevalence in historical reports varies between 0% and 42%. Symptoms of PVS are nonspecific and can include general symptoms such as dyspnea, cough, recurrent pneumonia, and chest pain. Pathophysiologically it increases the postcapillary pressure in the pulmonary circuit and may result in pulmonary hypertension (PH). Misdiagnosis and delayed treatment are common. We here report a case of a 59‐year‐old female with a history of pulmonary vein ablation followed by progressive dyspnea (New York Heart Association IV), right heart failure, CPR, and the need for extracorporeal membrane oxygenation (ECMO). Further treatment strategy includes pulmonary vein dilatation and stenting of both the left superior pulmonary vein and left inferior pulmonary vein, as well as balloon dilatation of RIPV under temporary ECMO support. Symptomatic, severe PVS is a rare complication after catheter ablation of atrial fibrillation. PVS can result in life‐threatening complications such as PH with acute right heart failure. Early diagnosis is crucial but challenging. Mechanical cardiopulmonary support by veno‐arterial ECMO for bridging to angioplasty could be a lifesaving option.

**Abbreviations**
AF: atrial fibrillation
BNP: brain natriuretic peptide
CPR: cardiopulmonary resuscitation
CT: computer tomography
LIPV: left inferior pulmonary vein
LSPV: left superior pulmonary vein
PH: pulmonary hypertension
PV: pulmonary vein
PVS: pulmonary vein stenosis
RSPV: right superior pulmonary vein
RV: right ventricle
V‐VA‐ECMO: veno‐veno‐arterial extracorporeal membrane oxygenation
VA‐ECMO: veno‐arterial extracorporeal membrane oxygenation

## BACKGROUND

Pulmonary hypertension (PH) due to pulmonary vein stenosis (PVS) following percutaneous PV isolation for atrial fibrillation (AF) is a serious but rare complication. Symptoms of severe PVS may vary from mild dyspnea to life‐threatening circulatory deterioration.^1^ Histopathological findings are characterized by fibrosis and scarring of the PV and proliferation of elastic laminae resulting in the reduction of the PV diameter and stenosis.^2^ The prevalence of PVS following PV isolation reported in the literature from 1999 to 2004 ranges from 0% to 42%–44%.^3^, ^4^ Recent studies after 2004 report a decrease in prevalence from 0% to 19% (mean: 2%; median: 3.1%).^4^ The incidence of severe PVS following PV isolation is approximately between 0.32% and 3.4%.^5^ Delaying diagnosis may allow progression of stenosis, resulting in PV occlusion causing severe damage of intraparenchymal lung tissue and microvasculopathy with a consecutive postcapillary PH leading to right heart failure. However, data regarding the long‐term outcome in patients with PVS with a need for intervention are sparse.

## CASE PRESENTATION

**Figure 1:**
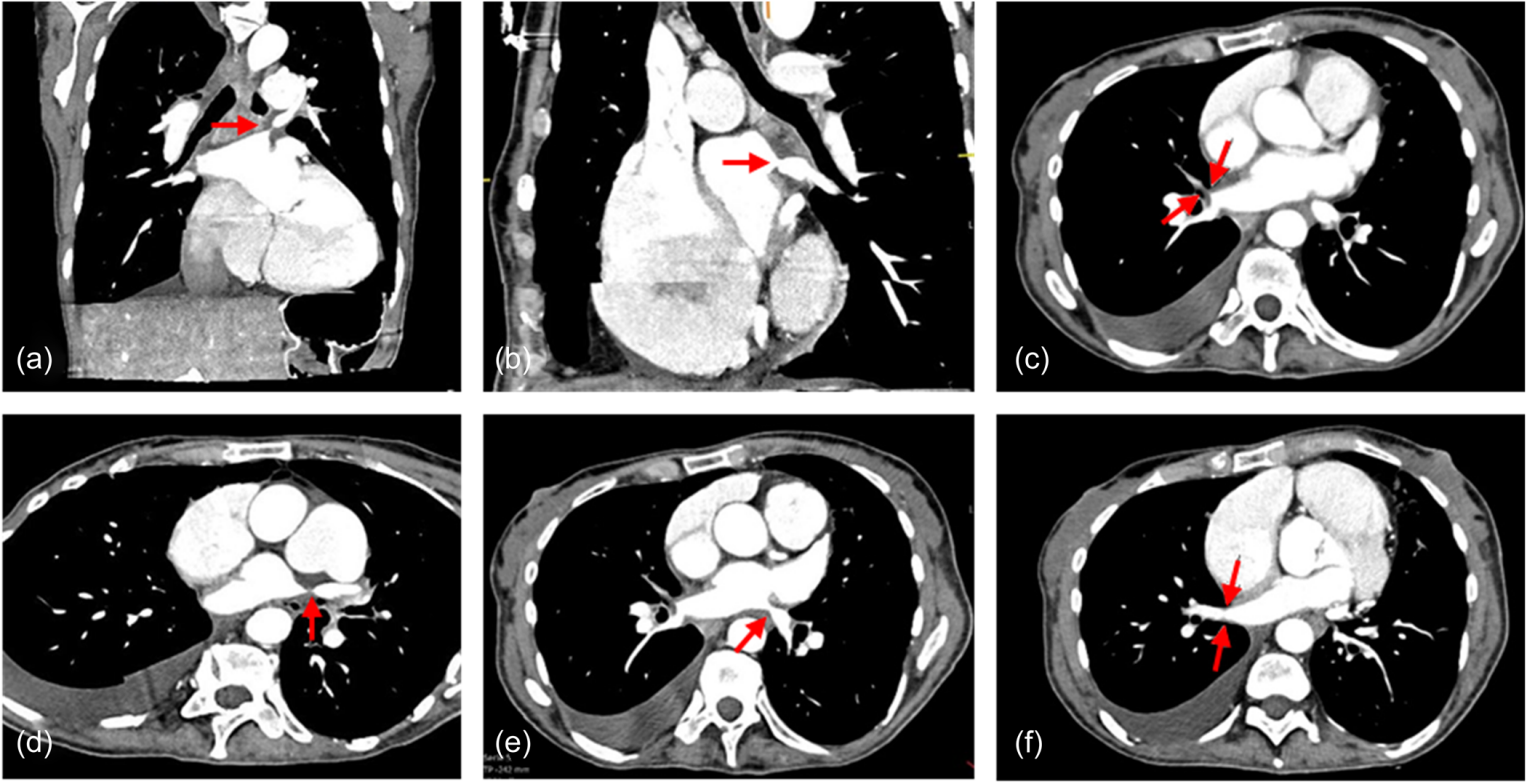
CT angiography at diagnosis of pulmonary vein stenosis. (a, d) Total occlusion of LSPV. (b, e) Stenosis of LIPV. (c) Total occlusion of RSPV in segment 3. (f) Stenosis of RIPV. CT, computed tomography; LIPV, left inferior pulmonary vein; LSPV, left superior pulmonary vein; RIPV, right inferior pulmonary vein; RSPV, right superior pulmonary vein.

**Figure 2:**
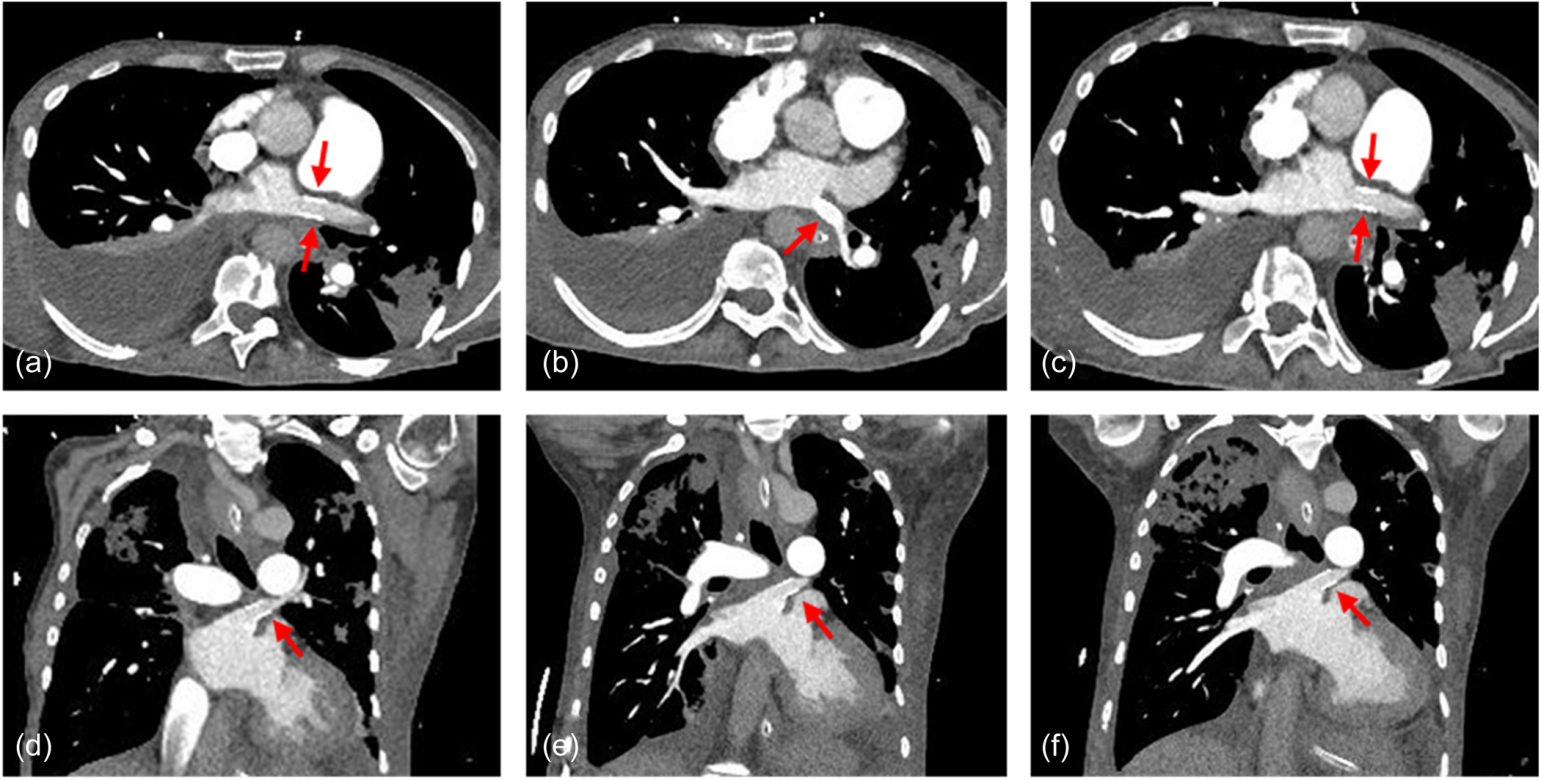
CT angiography at follow‐up examination 4 months after the clinical course. (a, d) Successful stenting of LSPV. (b, e) Successful stenting of LIPV. (c, f) Successful balloon dilatation of RIPV. CT, computed tomography; LIPV, left inferior pulmonary vein; LSPV, left superior pulmonary vein; RIPV, right inferior pulmonary vein.

A 59‐year‐old female patient presented with progressive dyspnea (New York Heart Association III–IV), general fatigue, and long‐term oxygen therapy at our outpatient clinic for PH. One year before, the patient was successfully treated with percutaneous PV isolation by radiofrequency ablation for AF. Approximately 7 months later, a follow‐up showed a peak tricuspid regurgitation velocity of 4.1 m/s and dilatation of the right atrium (31 cm^2^) and ventricle (right ventricular [RV] basal end‐diastolic diameter 48 mm), as well as pleural effusion, indicating a high clinical probability for the presence of PH according to the current 2022 ESC/ERS Guidelines for the diagnosis and treatment of PH.^6^ Cardiovascular computed tomography (CT) excluded the presence of coronary heart disease as underlying causes but demonstrated severe PVS secondary to the ablation procedure with relevant stenosis of all four PVs. The left superior PV (LSPV) and segmental branch of the right superior PV were totally occluded, and the inferior PVs presented with stenosis (Figure 1). Additionally, signs of chronic thromboembolic disease were present, including wall‐adherent thrombi, mosaic perfusion, and disparities in segmental vessel size. Consequently, right heart catheterization (RHC) was performed as the gold standard for diagnosing and classifying PH. RHC showed a combined pre‐ and postcapillary PH (mean pulmonary arterial pressure 42 mmHg/systolic pulmonary arterial pressure 72 mmHg/diastolic pulmonary arterial pressure 24 mmHg/pulmonary arterial wedge pressure 16 mmHg/pulmonary vascular resistance 4 WU/diastolic pulmonary gradient 8 mmHg). Laboratory results revealed signs of acute decompensated heart failure with brain natriuretic peptide (BNP) elevation of 2.373 pg/ml (ref.: <100 pg/ml) and troponin I 69.5 pg/ml (ref.: <24 pg/ml), creatinine kinase within the normal range (51 U/l). C‐reactive protein was slightly elevated (116 mg/l; ref.: <5 mg/l). Blood count and renal function were in the normal range. The initial treatment with intravenous diuretics did not result in the improvement of symptoms. A bilateral interstitial pneumonia was demonstrated, and antibiotic therapy was initiated. Three days later, the patient deteriorated into cardiogenic shock (hypotension, sinus tachycardia, impaired peripheral oxygenation, centralization). At this time and in the context of the limited conservative treatment options of acute on chronic right heart failure, introducer cannulas were placed percutaneously by Seldinger technique in the right femoral artery and vein preemptively expecting the potential need for extracorporeal membrane oxygenation (ECMO) support. Within this procedure, the patient developed lactate acidosis, nonsustained ventricular tachycardia, and hemodynamic fragility, resulting in an in‐hospital cardiac arrest with asystole. Cardiopulmonary resuscitation (CPR) was performed for 2 min. Shortly later, the patient had an additional episode of asystole and CPR for another 4 min and endotracheal intubation was performed. In the first return of spontaneous circulation (ROSC), a decision for the temporary use of mechanical circulatory support by veno‐arterial ECMO (V_f_‐A_f_d‐ECMO, Cardiohelp; Getinge) as a rescue procedure for bridging to angioplasty was made. Venous cannulation was performed using a 23 F/55 cm cannula (Maquet; Getinge) and arterial cannulation was performed using a 15 F/23 cm cannula (Maquet; Getinge). An additional arterial cannula distal to the femoral artery cannula for perfusion of the limb was established. Initial revolutions per minute were 4110 rpm generating 4.5 L/min ECMO blood flow. One day after initiation of V_f_‐A_f_d‐ECMO, oxygenation worsened because of progressive pneumonia. Massive pleural effusion on the right side impaired the oxygenation therefore a thoracentesis was performed. Arterial blood gas analysis showed hypoxemia (partial pressure of oxygen [paO_2_]: 56 mmHg) requiring a high concentration of inspired oxygen and positive end‐expiratory pressure (FiO_2_ 70%, positive end‐expiratory pressure 10 mbar). PaO_2_/FiO_2_ (P/F) ratio was 80 mmHg, which suggested severe hypoxemia. The decision for ECMO support was made and therapy was escalated with an additional venous cannula (19 F/15 cm) inserted into the right jugular vein for V_f_‐A_f_dV_j_‐ECMO. Separate monitoring of the returning blood flows of the jugular and the femoral cannula was performed by using an ultrasonic flow computer (SonoTT; medicovation). Temporarily, the venous return was controlled by compression of the venous cannula. The patient's left ventricular systolic pump function was always preserved. Both echocardiographic aortic valve opening and pulse pressure were measured adequately. Four days after cannulation, the patient was transferred to a center specialized in angioplasty of the PV. Since PV dilatation and stenting of LSPV and left inferior PV, as well as balloon dilatation of RIPV, was performed successfully (Figure 2), the intervention of the right superior segmental PV failed. After retransferring to our hospital, the clinical course improved, particularly the reduction of artificial ventilation up to the explantation of the V_f_‐A_f_dV_j_‐ECMO. Neurological examination revealed an alert patient with preserved communication. After tracheostomy and placement of a percutaneous gastroscopy tube, the patient was transferred to a weaning clinic for recovery.

During the first follow‐up examination and 4months after clinical worsening with V_f_‐A_f_dV_j_‐ECMO implantation, CT angiography was performed and showed successful stent implantation and angioplasty and absence of restenosis. Those results were also reflected by functional, clinical, and laboratory parameters. The patient improved from World Health Organization (WHO) functional Class IV to WHO functional Class II without the need for oxygen support. In addition, the patient increased the 6‐min walking distance from 230 to 300 m and a reduction of BNP from 1.296 to 123 pg/ml.

Further follow‐up of the patient including CT angiography of PVs will be mandatory to exclude PV restenosis, particularly with regard to the development of clinical symptoms related to potential PV restenosis.

## DISCUSSION AND CONCLUSIONS

Our reported case demonstrates PVS as a severe complication of radiofrequency ablation for therapy of AF with a life‐threatening course due to PH and consecutive acute fulminant right heart failure. To our knowledge, this case is the first report of a patient with a history of percutaneous PV isolation, who suffered from acute right heart failure followed by cardiogenic shock and need for VA‐ECMO as a bridging treatment to PV angioplasty. Few case series on patients with occlusion of PV treated by recanalization have been published.^7^ However, the symptoms in these three reported patients consisted of cough, hemoptysis, and dyspnea only. The incidence of PVS‐related symptoms vary depending on the severity of stenosis, the number of affected PV, and clinical circumstances.^1^ One study including 124 patients with severe PVS after AF ablation showed that clinical progression appeared 4 ± 3 months after the procedure. The mean delay between the first onset of symptoms and the final diagnosis of PVS was 4.4 ± 5.4 months later.^5^ Another large cohort study including 976 patients undergoing ablation reported a lower incidence of severe PVS (<1%) with a negligible need for intervention for symptomatic PVS.^8^ Necessity for treatment of severe, symptomatic PVS decreased from 0.71% (1995–2003) to 0.29% (2003–2006) until 0.1% (2005–2016) of patients who underwent AF ablation.^8^, ^9^

PVS can result in life‐threatening complications with PH and right heart failure. In patients with circulatory collapse or cardiac arrest, the use of a temporary support by VA‐ECMO can be recommended when initial treatment failed, and further treatment options can be performed under mechanical circulatory support (“bridge to treatment”). A recent study demonstrated that patients with CPR benefit either from VA‐ECMO alone or in combination with other treatment strategies if hemodynamic compromise due to right heart failure persisted.^10^ Thus, early diagnosis is crucial but challenging. Symptoms of PVS are nonspecific and general for pulmonary diseases. Misdiagnosis is common, leading to delayed treatment and recovery.^1^

However, frequent follow‐ups of the patient are necessary to recognize possible PV restenosis or worsening of the symptoms due to PH consistent with progressive damage of intraparenchymal tissue. After all, the risk for restenosis after stenting seems to reduce the risk of subsequent PV restenosis in comparison with balloon angioplasty (BA). Nevertheless, the 3‐year overall rate of restenosis of veins initially treated with BA is 49% and 25% in initial stenting corresponding to an increased risk of restenosis with BA versus stenting with a hazard ratio of 2.77 (95% confidence interval: 1.7–4.45; *p* < 0.001).^5^

Considering AF as the most common cardiac arrhythmia with a need for treatment awareness for the risk of PVS due to ablation is important. Future investigations to evaluate the occurrence of PVS depending on variables such as the used ablation method and delivered energy is needed.

## AUTHOR CONTRIBUTIONS

Material preparation, data collection, and the first draft of the case report were performed by Sabrina Kopp and Marie‐Kristin Tilch. All authors read and approved the final manuscript.

## CONFLICTS OF INTEREST

Marie‐Kristin Tilch received speakers' honoraria from Astra Zeneca, Shire Takeda, Hexal outside the submitted work. Malte Kuniss received consulting and speakers' honoraria from Medtronic. Kai‐Helge Schmidt, Thomas Rostock, Thomas Münzel, Johannes Wild, and Stavros Konstantinides report institutional grants and personal lecture/advisory fees from Bayer AG, Daiichi Sankyo, and Boston Scientific; institutional grants from Inari Medical; and personal lecture/advisory fees from MSD and Bristol Myers Squibb/Pfizer. Lukas Hobohm reports lecture/consultant fees from MSD and Actelion, outside the submitted work. The remaining authors declare no conflicts of interest.

## ETHICS STATEMENT

Written informed consent was obtained from the patient for publication of this case report and any accompanying images. A copy of the written consent is available for review by the Editor‑in‑Chief of this journal.

## Data Availability

All data generated or analyzed during this study are included in this published article.
